# Training Universal Deep-Learning Networks for Electromagnetic Medical Imaging Using a Large Database of Randomized Objects

**DOI:** 10.3390/s24010008

**Published:** 2023-12-19

**Authors:** Fei Xue, Lei Guo, Alina Bialkowski, Amin Abbosh

**Affiliations:** School of Electrical Engineering and Computer Science, The University of Queensland, Brisbane 4072, Australia; l.guo3@uq.edu.au (L.G.); alina.bialkowski@uq.edu.au (A.B.); a.abbosh@uq.edu.au (A.A.)

**Keywords:** database, deep learning, electromagnetic imaging, antenna sensing

## Abstract

Deep learning has become a powerful tool for solving inverse problems in electromagnetic medical imaging. However, contemporary deep-learning-based approaches are susceptible to inaccuracies stemming from inadequate training datasets, primarily consisting of signals generated from simplified and homogeneous imaging scenarios. This paper introduces a novel methodology to construct an expansive and diverse database encompassing domains featuring randomly shaped structures with electrical properties representative of healthy and abnormal tissues. The core objective of this database is to enable the training of universal deep-learning techniques for permittivity profile reconstruction in complex electromagnetic medical imaging domains. The constructed database contains 25,000 unique objects created by superimposing from 6 to 24 randomly sized ellipses and polygons with varying electrical attributes. Introducing randomness in the database enhances training, allowing the neural network to achieve universality while reducing the risk of overfitting. The representative signals in the database are generated using an array of antennas that irradiate the imaging domain and capture scattered signals. A custom-designed U-net is trained by using those signals to generate the permittivity profile of the defined imaging domain. To assess the database and confirm the universality of the trained network, three distinct testing datasets with diverse objects are imaged using the designed U-net. Quantitative assessments of the generated images show promising results, with structural similarity scores consistently exceeding 0.84, normalized root mean square errors remaining below 14%, and peak signal-to-noise ratios exceeding 33 dB. These results demonstrate the practicality of the constructed database for training deep learning networks that have generalization capabilities in solving inverse problems in medical imaging without the need for additional physical assistant algorithms.

## 1. Introduction

Electromagnetic (EM) imaging has emerged as a versatile, cost-effective, and non-invasive modality applicable to diverse domains such as medical diagnosis, infrastructure inspection, security, and various imaging and sensing fields [1,2,3]. EM imaging entails reconstructing an imaged object’s electrical properties from the measured scattered field as an inverse problem, known for its ill-posed nature [4,5]. Traditionally, inverse scattering algorithms employ either iterative or noniterative methods for calculating the electrical properties of imaged objects. Noniterative methods often rely on impractical assumptions that do not suit real-life applications. In contrast, iterative methods [6,7,8] exhibit the drawback of prolonged computation times, rendering them unsuitable for emergent scenarios like stroke detection [9].

As a contemporary alternative, deep learning offers rapid solutions [10,11] with the potential for enhanced imaging quality when adequately trained [12,13]. To establish a robust neural network for deep learning EM imaging, extensive training on a database that encompasses valid propagation or scattering models is imperative. Such a database typically comprises source data (i.e., time-domain or frequency-domain signals) and their corresponding labels (i.e., electrical property distributions of the imaged object) [14]. Acquiring source data in clinical EM imaging poses challenges due to the need for data from numerous subjects, and obtaining corresponding labels necessitates using complementary imaging modalities like magnetic resonance imaging or computed tomography. Consequently, the other feasible approach involves using simulated data for training, pretesting, and fine-tuning the neural network. The database construction process consists of designing electrical property distributions for imaged objects and capturing time-domain or frequency-domain signals scattered from these objects [15]. Such signals can be computed using established computational EM imaging techniques such as the finite-difference-time-domain (FDTD) or the finite-element-method (FEM). Furthermore, deep learning EM imaging algorithms may employ conventional tomography algorithms, including born iterative methods [16,17,18], contrast source [19,20], subspace [21], Gauss−Newton [22,23], and gradient methods [9,24,25] to generate initial estimates of imaged objects’ electrical properties.

The MNIST dataset [26] of handwritten digits has been frequently used for training deep neural networks in electromagnetic imaging of simple objects [27]. However, practical applications like medical imaging often involve complex structures. Therefore, databases with simple, single objects like MNIST may not be ideal for training and testing neural networks. Standard databases containing more complex objects, such as MS-COCO [28], ImageNet [29], and CIFAR-10 [30], are generally used for computer vision tasks. Nevertheless, the characteristics of objects in these databases significantly differ from those encountered in medical applications. Consequently, the transformation of extensive datasets into electromagnetic signals remains a challenging endeavor for training viable neural networks in medical imaging

Open-access clinical image databases [31,32,33,34,35] based on magnetic resonance imaging and computed tomography are not suitable for training data-driven neural networks for EM imaging. These databases require intricate image segmentation and precise assignment of electrical constants to various tissues, leading to the time-consuming generation of FDTD EM signals. Additionally, these databases are often categorized by specific anatomical body regions, which limits the applicability of networks trained on one region to other anatomical areas. For example, a network trained on a database of brain images may struggle to be applied to breast imaging effectively. Hence, insufficient data remains a common issue in medical EM imaging applications.

Inadequate dataset diversity can result in overfitting, compromising network performance. Thus, the need for a standard and more accessible approach to quickly building databases becomes apparent for training a generalized neural network. Researchers have recently investigated the impacts of estimating tissue microstructure from different training data distributions [36], highlighting that estimating model parameters using supervised machine learning depends strongly on the training-set distribution. An extensive multirandomly shaped graph database trained in a deep convolutional network [37] can utilize single-orientation phase data without secondary model training and successfully deliver quantitative susceptibility maps. Numerous data augmentation solutions have been reviewed in [38] to avoid overfitting in deep learning models on limited data applications. Although the physical imaging mechanisms of these works are different from the electromagnetic imaging principles discussed in this paper, the method of establishing a training dataset is instructive for this paper. Therefore, constructing a comprehensive, readily achievable database with vast diversity is essential to ensure the applicability of the trained neural network in a broader range of imaging tasks.

This paper introduces an approach to building a diverse database for two-dimensional (2D) electromagnetic imaging. The database comprises 25,000 distinct cases formed by superimposing 6 to 24 objects with random geometries and electrical properties. For example, all objects are within an imaging area defined by a 130-mm-radius circle. Sixteen antennas evenly distributed on the perimeter of a 150-mm-radius circle serve as signal transmitters and receivers, generating 256 sets of signals for each case through a custom 2D-FDTD solver. In addition, we chose a mature deep network structure to implement deep neural networks for high-precision electromagnetic imaging. While the commonly used deep neural networks, such as AlexNet [39], VggNet [40], ResNet [41], DenseNet [42], GoogleNet [43], and U-net [44], may all have promising performance for EM image reconstruction, we selected one of these networks (U-net) as the baseline model to objectively compare the generalization performance of the trained network with established databases.

As one of the deep learning networks, U-net has an encoder−decoder architecture, which achieves higher accuracy for image segmentation and is widely used in medical imaging applications. Hence, a tailoring U-net-based deep neural network is trained and tested on random objects generated via superimposed geometries and the MNIST dataset to evaluate the constructed database. The performance of the neural network trained on the proposed database is compared with that trained on the MNIST database, revealing enhanced universality in imaging various object shapes.

In summary, the contributions of this work are the following:

- A convenient and scalable method to generate a database encompassing objects with random shapes that have the electrical properties of human tissues is proposed for training and testing deep-leaning-based EM imaging methods. Our approach can be easily implemented to enlarge the database when larger training data is needed.
- A U-net-based EM imaging method that can reconstruct the permittivity profiles of tissues is proposed. This work is the first attempt to use complex multigeometrical objects to build training data that can be used to improve the universality of data-driven deep learning-based EM imaging without using physical algorithms.
- Using three different dataset testing results, the importance of a highly randomly shaped object database to achieve universal results in a data-driven EM imaging assessment is demonstrated. Given equal training epochs, training U-net with the database generated by our method outperforms the traditionally used MINST database. This work highlights the improved versatility of trained U-net using our diverse database.

The subsequent sections provide a detailed description of the constructed database (Section 2), the U-net models employed for showcasing the database’s utility (Section 3), present the results (Section 4), and conclude the paper (Section 5).

## 2. The Mixed Geometric Objects Database

### 2.1. Data Preparation

**Figure 1:**
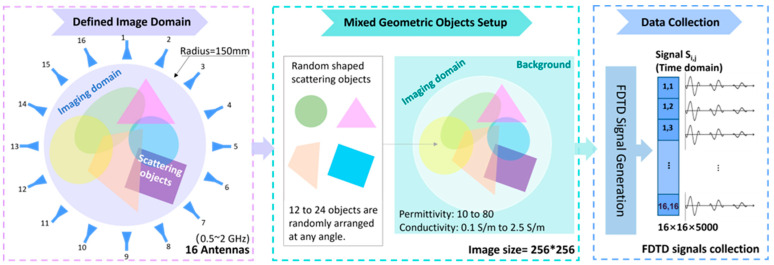
The workflow for building an imaging domain with mixed geometric objects and collecting EM data.

Given the intricate nature of the human tissues and the diverse range of medical imaging applications, the construction of a suitably extensive dataset encompassing a variety of geometric objects for deep neural network training was carried out in three stages: 1. defining the imaging domain; 2. creating and mixing random geometric objects; and 3. simulating the imaging domain and collecting the data. These steps are explained in Figure 1.

As an example, a circular area with a radius of 150 mm is defined as the imaging domain, which is suitable for most EM medical imaging applications, e.g., breast imaging, head imaging, knee imaging, etc. Sixteen Hertzian antennas that operate as transmitters and receivers are evenly distributed around the domain on the perimeter of a circle with a radius of 150 mm. Considering the limited system space, this number of antennas is particularly feasible for microwave medical imaging. A multistate scheme is used to transmit and receive the signals.

For the universality of the database, an object is formed in the imaging domain via superimposition of 6 to 24 geometries with different dimensions and properties. The geometries include ellipses with random long axes and short axes varied between 50 mm and 120 mm and polygons with various numbers of edges ranging from three to six. Each of the geometries is rotated with a random angle range from $0^{{}^{\circ}}$ to $360^{{}^{\circ}}$, and then superimposed to form randomly shaped structures. When configuring the antenna signals with the specified frequency, the imaging domain is filled with random values for the relative permittivity ( ε ) and conductivity (σ S/M) ranging from 10 to 80 and 0.2 to 2.5 S/m, respectively, which represent most human tissues [45], including but not limited to fat (ε = 10–15, σ = 0.08–0.2), bone (ε = 11–21, σ = 0.1–0.3), skin (ε = 38–45, σ = 0.7–1.2), brain tissues (ε = 45–53, σ = 1.5–1.8), cerebrospinal fluid (ε = 66–70, σ = −2.28–3.0), etc. During the superposition of multiple graphical regions, the permittivity and conductivity values are scaled to maintain values within the defined interval for the imaging region. The electrical properties of the region between the imaging domain and antennas are filled with material that has a dielectric contact and conductivity of 40 and 0.1 S/m, respectively, to emulate the commonly used matching medium in most EM medical imaging applications.

Once the imaging domain is constructed, an in-house built 2D-FDTD solver is used to simulate the scattered time-domain signals using a mesh size of 2 mm×2 mm and a time step of 4.72 picoseconds, resulting in a total simulation time of 23.6 ns (5000 time steps). Considering the tradeoff between strong tissue penetration of low frequencies and fine image resolution of high frequencies, we utilized a Gaussian pulse with a 10 dB bandwidth ranging from 0.5 to 2 GHz as the signal. This bandwidth is deemed suitable for most EM medical imaging applications [16]. A perfectly matched layer boundary condition truncates the simulation domain. The size of the entire simulation domain is 500 mm×500 mm. A multistate configuration is used in the simulations, so the simulated time-domain signals have the size of 16×16×5000.

### 2.2. Training and Test Datasets

**Figure 2:**
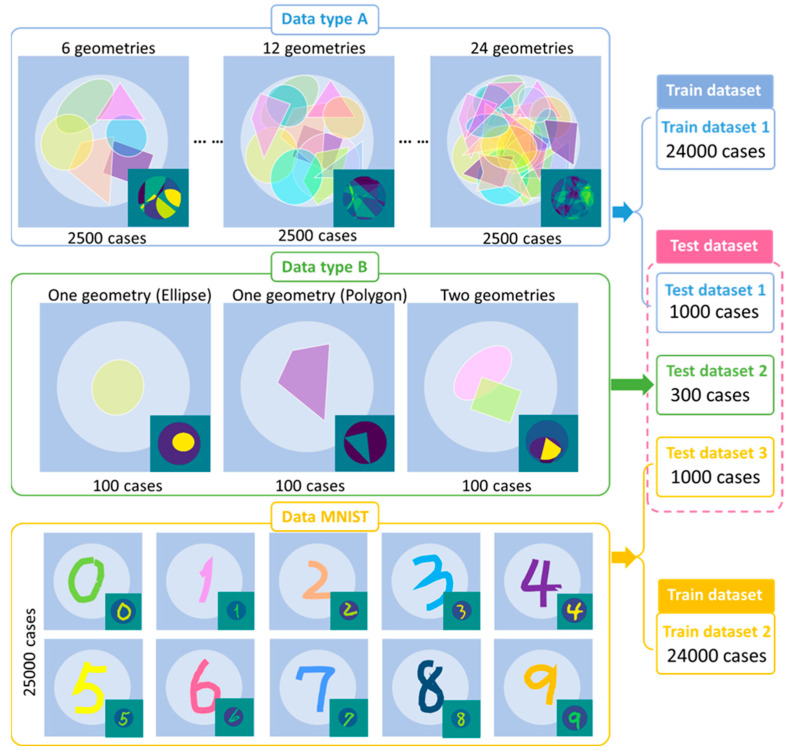
Database composition and geometry settings in the imaging domain.

Figure 2 illustrates the database composition and geometrical settings in the imaging domain as used in training and verification. The dataset—Data A—includes 25,000 cases with the electrical property profiles and their corresponding time domain signals. It is divided into ten groups with the same number of cases, but the superimposed geometries are increased from 6 to 24 different random ellipses and polygons. Among Data A, 24,000 shuffled cases are used as the training dataset, while the remaining 1000 cases are used as the testing dataset for Data A. To prove that the constructed database can improve the universality of a trained neural network, another testing dataset denoted as Data B is composed of 300 simple geometries (one single ellipse, one polygon, or two geometries), and their corresponding time-domain signals are also included as the source data. In addition, the MNIST database is also used to compare with the proposed database (Data A and B). A total of 25,000 handwritten digits are assigned with random relative permittivity and conductivity ranging from 10 to 80 and 0.2 to 2.5 S/m, respectively. The same FDTD algorithm is used to generate the time domain signals. Out of these, 24,000 cases were utilized for training the neural network, while the remaining 1000 were used for testing.

The time-domain signals are simulated using the 2D-FDTD solver in MATLAB. The FDTD simulation for each case takes 2.3 min by using the AMD EPYC Milan series processors equipped in the high-performance computing clusters at the University of Queensland.

## 3. Deep Learning Structure for Image Reconstruction

### 3.1. Training and Testing Procedure

**Figure 3:**
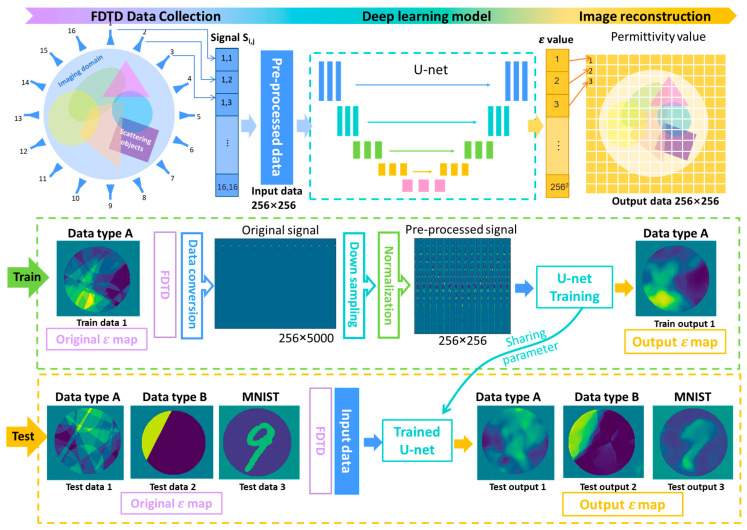
The procedure of DL-based permittivity profile reconstruction and the strategy for training and testing.

As shown in Figure 3, the procedure of deep learning-based permittivity profile reconstruction includes three steps. First, in the FDTD data collection step, based on the FDTD algorithm, the raw data of 16 × 16 time-domain signals with 5000 time samples were calculated. Then, the 16 × 16 × 5000 tensor data is processed to 256 × 256 input data by applying data preprocessing methods, including conversion, down-sampling, and normalization. The processing details can be seen in the training process in Figure 3. Then, in the second step, the preprocessed input data is used to train a U-net-like neural network to reconstruct the electrical property values. Finally, the trained U-net can learn to rebuild a 256 × 256 pixels image showing the permittivity profile value from the input data.

In the training process (the green dotted box of Figure 3), a total of 24,000 cases in Data A were used to train the proposed U-net, and the U-net learned to reconstruct the image of Data A, which consists of the randomly shaped multi-objects. In the testing process (the yellow dotted box of Figure 3), the remaining 1000 cases from Data A, 300 cases from Data B (randomly shaped single or double objects), and 300 cases from the MNIST (handwritten digit) dataset were used to test the U-net that trained by using the proposed database (Data A).

### 3.2. Data Preprocessing and Network Architecture

**Figure 4:**
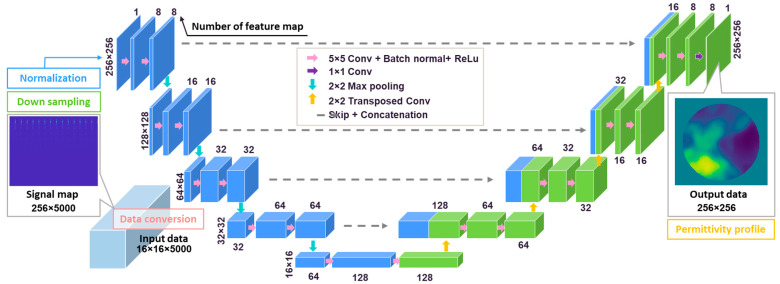
The architecture of the U-net is used in DL-based permittivity reconstruction.

The developed deep neural network is based on a modified version of the well-known U-net [44]. Figure 4 shows the structure of the proposed neural network. First, the 16 × 16 × 5000 tensor signals from all the transmitter and receiver pairs are stacked to form a signal map with the size of 256×5000. Second, the length of the time-domain signal is downsampled from 5000 to 250 using a downsampling rate of 20. Then, the signals are further padded with six columns of 0, and the final size of the input tensor is 256×256. We considered the inconsistent range of the response signals from different antenna pairs, as some signals from distant antenna pairs are too weak to be sensed by the neural network. So, before the input data are applied to the U-net, we processed the input tensor with a normalization operation named Min−Max feature scaling, which can be formulated by:

(1) $$X^{\prime }\left(n\right)=\frac{X\left(n\right)-X_{min}\left(n\right)}{X_{max}\left(n\right)-X_{min}\left(n\right)}$$

where $n\in \left[1,\;256\right]$ denotes the channel of the input tensor. $X\left(n\right)$ is the original signal in the nth channel of the input tensor. Here, $X_{max}\left(n\right)$ and $X_{min}\left(n\right)$ are the max and minimum values of the nth channel of the input tensor. $X’\left(n\right)$ is the final normalized signal.

The U-net architecture can be divided into encoding and decoding parts. The encoding part is used to extract the data features, while the decoding part is used to recover the image. The U-net has a depth of five, and each level consists of two convolutional layers with a kernel size of 5 × 5, a stride length of 2 × 1, and rectified linear units (ReLU). Furthermore, the max-pooling layers are added and halve the size of the previous input data size. The channel numbers of feature maps increase from 8 to 128, and the size of the bottom feature map is 16 × 16.

In the decoding part, the neural network consists of transposed convolutional layers, which include filters of size 2 × 2, a stride length of 2 × 2, and ReLUs. The convolutional layers in the decoding part are equivalent to those in the encoding part. As the channel numbers of feature maps decrease to 8, the signal map is finally transformed to a permittivity profile with a size of 256 × 256 by a 1 × 1 convolutional layer.

The adaptive moment estimation, “Adam” optimizer [46], is used to update the neural network parameters with a learning rate of 0.0005. In this case, the mean squared error is used as the loss function. The batch size is 32.

## 4. Results and Discussion

### 4.1. Testing Results of Permittivity Profile Reconstruction

**Figure 5:**
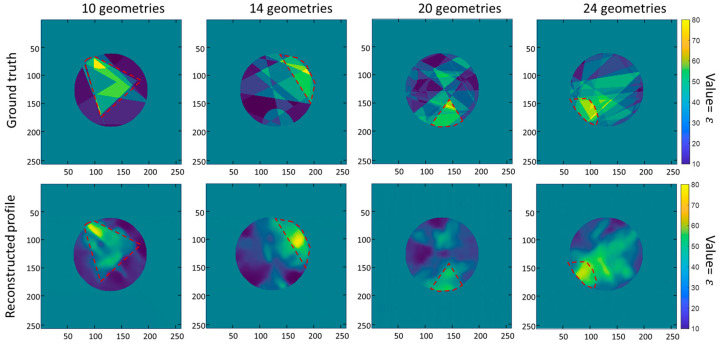
These are representative results of the proposed database-trained network in the Data A testing. The ground truths of scatterers and the reconstructed permittivity distributions are shown in the first and second rows, respectively. The red dotted box represents the expected regions with high permittivity values.

First, we demonstrate the experimental performance of the deep network trained by the proposed database (Data A). Some representative testing results for the objects superimposed by an excess of six geometries are shown in Figure 5. It shows the reconstructed permittivity value distributions for the superimposed geometries in the Data A test and their corresponding ground truth images. The red dashed lines represent the regions with high permittivity values. The reconstructed permittivity maps follow the distributions of the ground truth maps, in which the areas with high and low permittivity values are correctly reconstructed.

**Figure 6:**
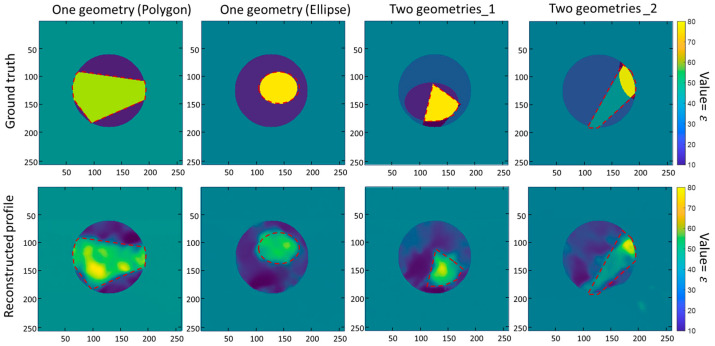
Representative results of the proposed database-trained network in the Data B testing. The ground truths of scatterers and the reconstructed permittivity distributions are shown in the first and second rows, respectively. The red dotted box represents the expected regions with high permittivity values.

Figure 6 shows the reconstructed permittivity distributions (Data B testing) for objects superimposed by one (the 1st and 2nd columns in Figure 6) and two (the 3rd and 4th columns in Figure 6) geometries and their corresponding ground truth images. The results demonstrate that the permittivity distributions can be well reconstructed, in which the regions with high/low permittivity values match well with the ground truth. These results also indicate that the constructed database has a positive impact on improving the universality of the trained neural network.

**Figure 7:**
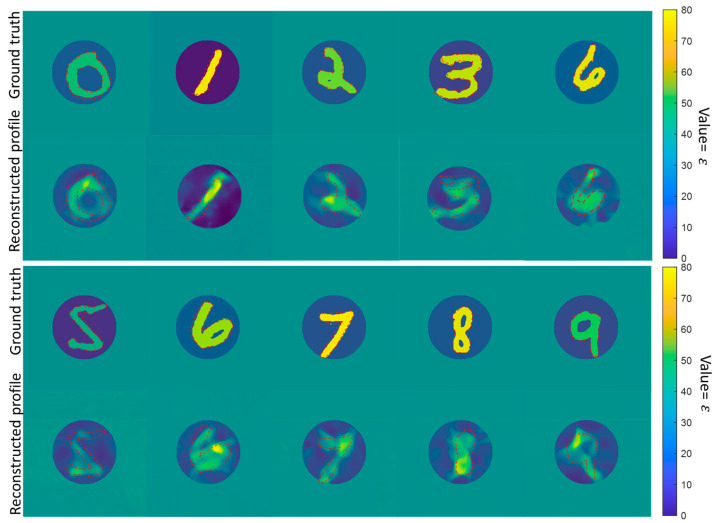
Representative results of the proposed database-trained network in the MNIST data testing. The ground truths of scatterers (1, 3 rows) and the reconstructed permittivity distributions (2, 4 rows) are shown, respectively. The red dotted box represents the expected digits imaging areas with high permittivity values.

Figure 7 shows the permittivity distributions for the objects constructed by the handwritten digits from zero to nine and their corresponding ground truth images. The results show that the permittivity distributions can also be well reconstructed to recognize the digits. Since the imaged objects are formed with handwriting digits, they significantly differ from the training dataset (Data A). Therefore, these results prove that the proposed database can substantially promote the universality of the trained networks.

As a comparison, the same network was trained by the MNIST database. The network trained by the MNIST database was also tested using Data A, B, and MNIST data separately. This comparison aims to find which database can improve the U-net’s universality when applied to a new database with significantly different data distribution.

**Figure 8:**
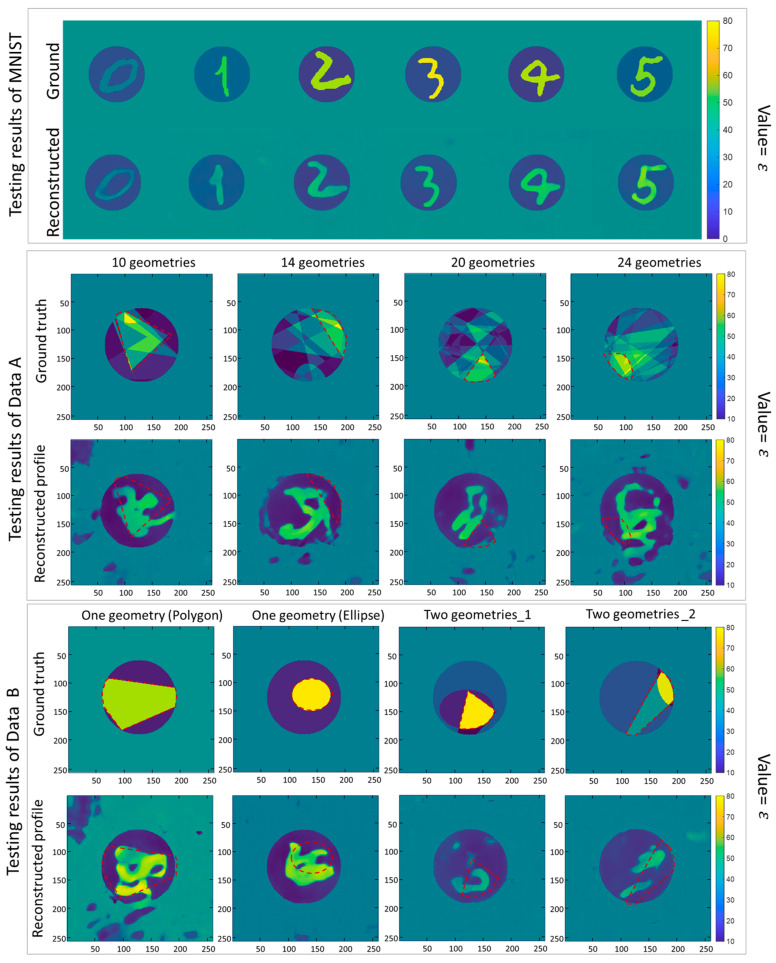
Representative results of MNIST-trained network in all the test datasets. The ground truths of scatterers (1, 3, and 5 rows) and the reconstructed dielectric distributions (2, 4, and 6 rows) are shown, respectively. The red dotted box represents the expected imaging areas with high permittivity values.

As shown in Figure 8, desirable results are achieved when the trained neural network is tested using the data from the MNIST database. However, when the trained network is tested on different datasets (Data A and B), the imaging results are significantly degraded.

The results show that the achievable performance of the network trained using the MNIST database is limited to datasets with highly similar data distribution. On the other hand, the neural network trained by the proposed database (Data A) sees an improved universality since it can achieve the required testing results even when using data with significant differences from the data used in training, such as testing on the MNIST data using a network trained using our EM database.

### 4.2. Assessment Metrics of Testing Results

To quantitatively evaluate the performance of the proposed database (Data A), three conventional assessment metrics are used: the structural similarity index measure (SSIM) [47], the normalized root mean square error (NRMSE), and the peak signal-to-noise ratio (PSNR) [48]. The SSIM is designed to predict the mismatch between reconstructed images (relative permittivity) and corresponding ground truths on the pixel level, and the higher (Range between [0, 1]) of the SSIM, the better. The NRMSE is the statistical value obtained after normalizing the root-mean-square error (RMSE), and the lower the values (Range [0, 1]) of the RMSE, the better. PSNR is another type of assessment metric to quantify reconstruction quality for images. The higher the PSNR, the smaller the error.

**Figure 9:**
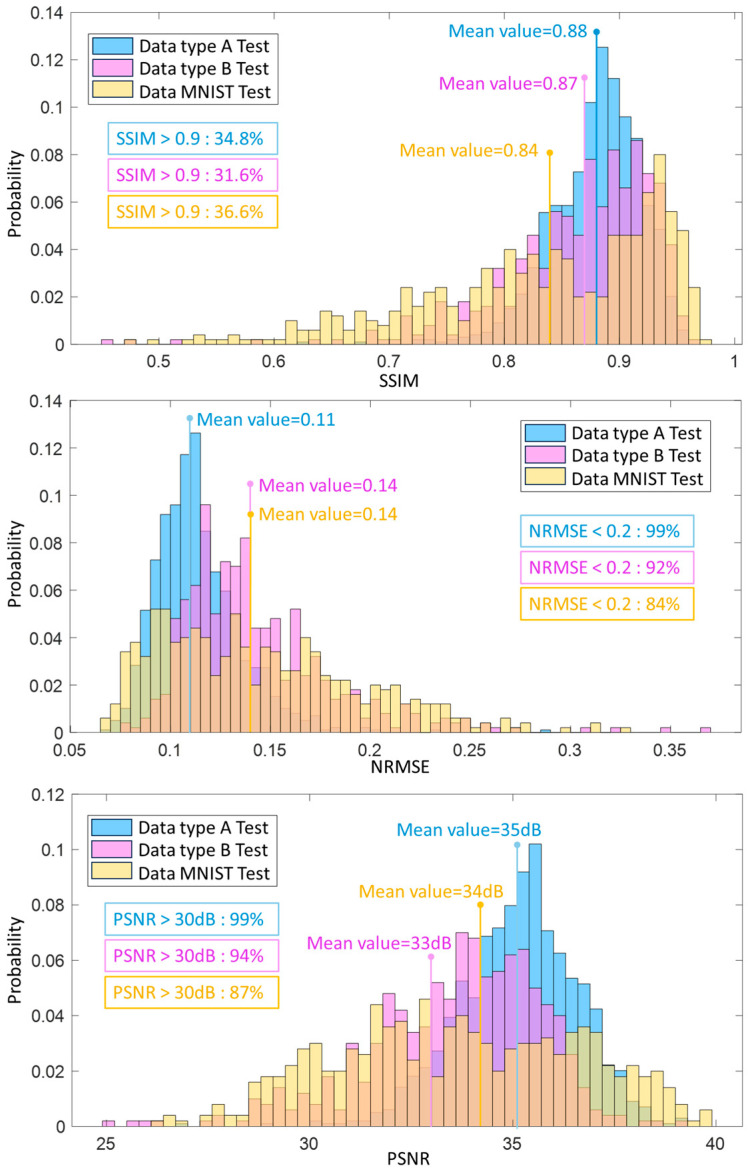
The evaluation histograms of Data A trained network using SSIM, NRMSE, and PSNR for all reconstruction profiles in both the test datasets. (Apart from the blue, pink and yellow bars, the bars in other colors are the overlapping parts of these three bars.)

The SSIM, the NRMSE, and the PSNR of the generated results from the neural network trained by the proposed database and tested by Data A, B, and MNIST data are given in Figure 9. The histograms of SSIM illustrate that all values in the testing datasets are between 0.8 and 0.95, which can be seen as a good quality score [47]. The proportions of SSIM values exceeding 0.9 in all the test datasets are relatively comparable, standing at 34.8%, 31.6%, and 36.6% for the respective sets. Notably, the mean SSIM values for these three test sets are also quite similar, at 0.88, 0.87, and 0.84, respectively. The histograms of NRMSE show that 99% and 92% of reconstructed permittivity in results in type A and B type testing datasets are less than 0.2.

In contrast, only 84% of testing results in the MNIST testing dataset is less than 0.2. The mean values of NRMSE in type B and MNIST testing datasets are the same at 0.14. The histograms of PSNR indicate that 99% of the results in the type A testing dataset are more than 30 dB (the baseline of accepted value [48]), and the PSNR values from the results of type B and MNIST testing datasets are 94% and 87% separately. The mean values of PSNR in the three testing datasets are close to 35 dB, 33 dB, and 34 dB, respectively.

The assessment metrics quantitatively illustrate that, no matter whether in Data A (with similar data distribution with training data), Data B (with a specific different data distribution), or even MNIST data (with entirely different data distribution), the proposed database trained network was able to reconstruct permittivity profiles with high quality.

**Figure 10:**
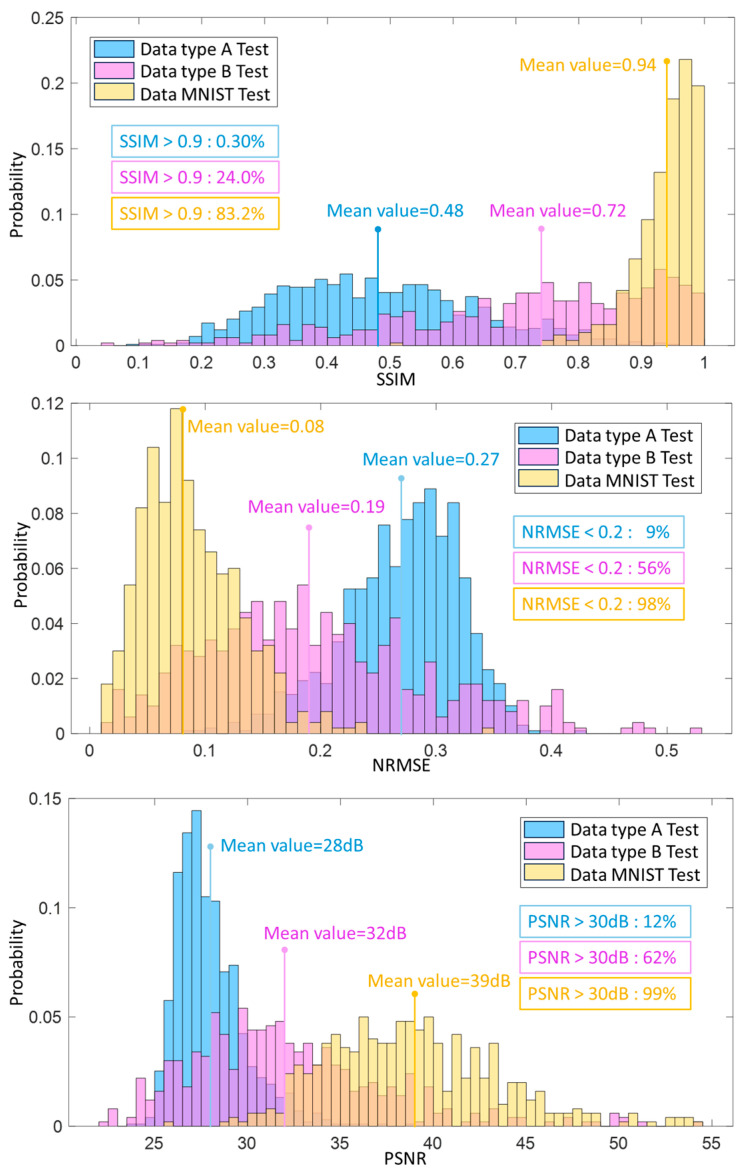
The evaluation histograms of the Data MNIST-trained network using SSIM, NRMSE, and PSNR for all reconstruction profiles in all the test datasets. (Apart from the blue, pink and yellow bars, the bars in other colors are the overlapping parts of these three bars).

On the other hand, we calculated the SSIM, the NRMSE, and the PSNR of the neural network trained by the data MNIST and compared them with the results from the neural network trained by the proposed database. The three evaluation metrics of testing on Data A, Data B, and Data MNIST based on the data MNIST-trained network are depicted in Figure 10. The histograms of SSIM are different from Figure 9. The SSIM results of the MNIST testing dataset indicate that 83.2% of testing cases have SSIM values higher than 0.9, and the mean value is 0.94. In contrast, the results from the other two testing datasets are pretty low. In particular, only 0.3% of the testing results in the type A testing dataset are higher than 0.9, and the corresponding mean value is 0.48. The histograms of NRMSE show the same biased tendency. Though the proportion of NRMSE, which is smaller than 0.2 in the MNIST testing dataset, is high at 98%, the proportion of the Data A testing dataset is relatively low at 9%. Similar to the first two evaluation metrics, the mean value of PSNR in the data MNIST test (39 dB) and the proportion of the acceptable value higher than 30 dB (99%) is pretty high. However, the mean of PSNR in the Data A test is just 28 dB, and its proportion of acceptable value is only 12%.

The detailed value of means, standard deviations of SSIM, NRMSE, and PSNR of all reconstructed test results between the comparison experiments, and the corresponding proportion that meets the requirements under the baseline are computed and shown in Table 1. The results of the comparisons (in the 3rd and 4th rows) show that the reconstruction performance of the Data A-trained network (test on Data MNIST) is better than the Data MNIST-trained network (test on Data A). It indicates that compared with the network trained by MNIST, the deep neural network trained by Data A, which is part of the built database in this paper, is not restricted.

### 4.3. Discussion

Based on the reported analysis, which combines both qualitative assessments (see Figure 5, Figure 6 and Figure 7) and quantitative evaluations (detailed in Table 1), it is clear that a network trained using the proposed database consistently generates high-quality and reliable images. This phenomenon remains true when the network is tested on different data types, including Data A, Data B, and the conventional MNIST dataset. However, suppose the network is trained using the MNIST dataset. In that case, it only delivers acceptable results when the test data is of the same MNIST type, and its performance significantly degrades when dealing with different domains. This observation underscores a significant advantage of the proposed database over the conventional MNIST dataset, especially in enhancing the adaptability of deep learning networks across various domains with diverse data distributions. This capability is especially crucial in medical imaging scenarios.

The results presented in this section collectively demonstrate that our multirandom geometrical database is better suited for training deep learning models, leading to superior generalization performance. This generalization ability can be effectively harnessed for reconstructing electromagnetic profiles for objects with unknown characteristics, such as medical imaging.

This work presents a methodology to construct training datasets that can generalize deep learning networks for medical microwave imaging applications. Most public datasets are either too narrow in imaging content, targeting computer vision applications, or too simple, making them inadequate for training medical imaging deep networks that can perform well in real-life scenarios. This work tries to overcome these challenges and opens up avenues for future research that can address its limitations. For example, this work can be expanded from using two-dimensional signals to three-dimensional signals that are more realistic. Moreover, while this work uses a 16-antenna system that is compatible with most microwave medical imaging systems, the methodology can be extended and applied to different antenna configurations depending on the specific applications.

## 5. Conclusions

A method is presented for constructing a comprehensive database to train deep-learning neural networks in electromagnetic medical imaging. The database improves the universal applicability of the trained deep learning network by deliberately introducing randomness and diversity in the shape, location, and dielectric properties. The proposed database is superior to existing databases, which were designed for other applications but are being used in electromagnetic imaging, such as MNIST. The predicted permittivity profiles of MNIST data reconstructed by the custom-built U-net trained by the presented data showed high-quality images with structural similarity index measure of more than 0.84, normalized root mean square error below 14%, and peak signal-to-noise ratio of more than 33 dB. In contrast, using U-net trained by MINST data resulted in unsatisfactory reconstructed profiles.

The proposed database is versatile and can be a valuable resource for researchers working on developing deep-learning methodologies for electromagnetic medical imaging, particularly in scenarios where medical data may be limited or where diverse shapes and structures need to be considered. The proposed database also holds potential as a pretraining dataset for future transfer learning and domain adaptation applications, especially as these algorithms are tested in real-world medical imaging settings.

## Figures and Tables

**Table 1:** The comparison between the two trained networks with the detailed means, the proportion of acceptable value, and the corresponding standard deviations of SSIM, NRMSE, and PSNR of all reconstruction profiles in the tested cases.

Train Dataset	Test Dataset	SSIM			NRMSE			PSNR (dB)		
		Mean	% >0.9	Std. *	Mean	% <0.2	Std.	Mean	% >30 dB	Std.
Data A	Data A	0.8777	34.8%	0.0391	0.1138	99.3%	0.0212	35.130	89.8%	1.4643
	Data B	0.8675	31.6%	0.0708	0.1434	92.4%	0.0382	33.600	94.3%	2.1125
	**MNIST**	**0.8357**	**36.6%**	0.1026	**0.1446**	**84.3%**	0.0499	**33.651**	**87.2%**	2.9745
MNIST	**Data A**	**0.4801**	**0.3%**	0.1552	**0.2712**	**9.3%**	0.0499	**27.971**	**12.1%**	1.8462
	Data B	0.7213	24.0%	0.2063	0.1953	56.2%	0.0954	32.066	61.8%	5.2021
	MNIST	0.9398	83.2%	0.0502	0.0883	98.2%	0.0429	38.809	99.0%	4.6237

* Std.: Standard deviation.

## Data Availability

The data in this paper can be obtained from the corresponding author.
